# Successful treatment of refractory classic juvenile pityriasis rubra pilaris with adalimumab in a 4-year-old girl: a case report

**DOI:** 10.3389/fimmu.2026.1885641

**Published:** 2026-08-14

**Authors:** Jianlan Zhang, Yuyue Zuo, Ruili Jiang, Yun Xia, Jinbo Chen

**Affiliations:** 1 Department of Dermatology, Wuhan No. 1 Hospital, Wuhan, Hubei, China; 2 Hubei Province & Key Laboratory of Skin Infection And Immunity, Hubei Provincial Clinical Research Center for Infectious Skin Diseases, Wuhan, Hubei, China

**Keywords:** adalimumab, autoinflammatory skin disease, juvenile PRP, pediatric immunology, pityriasis rubra pilaris, TNF-α inhibitor

## Abstract

**Background:**

Classic juvenile pityriasis rubra pilaris (PRP) (Griffiths type III) is a rare inflammatory papulosquamous dermatosis that is often refractory to conventional topical and systemic treatments. Despite the growing use of tumor necrosis factor-α (TNF-α) inhibitors in adult PRP, clinical evidence for their efficacy and safety in children under 5 years remains extremely limited.

**Case presentation:**

We herein report a 4-year-and-7-month-old Chinese girl presenting with a 20-day history of rapidly progressive generalized erythema, follicular keratotic papules, diffuse scaling, severe palmoplantar keratoderma and intractable pruritus. Initial differential diagnoses included psoriasis and pityriasis rubra pilaris (PRP), and skin histopathology confirmed classic juvenile PRP. Prior interventions with topical corticosteroids, emollients, oral antihistamines and traditional Chinese medicine yielded minimal clinical improvement. From the caregiver’s perspective, persistent extensive cutaneous lesions with unremitting pruritus disrupted the child’s daily activities, and multiple unsuccessful conventional therapies triggered considerable anxiety regarding long-term disease management. Given disease refractoriness, off-label adalimumab therapy was initiated. The patient received subcutaneous adalimumab at an initial dose of 20 mg at week 0, followed by 20 mg at week 1, and subsequent maintenance doses of 20 mg every two weeks, with a total of 8 injections. Approximately 3 months after treatment initiation, all cutaneous lesions achieved complete resolution, with only residual hypopigmented macules observed. No severe adverse events occurred during the treatment course. At the latest follow-up, 8 months after the final adalimumab injection, the patient maintained sustained complete remission without relapse.

**Conclusion:**

This clinical case demonstrates that adalimumab achieves rapid therapeutic efficacy and favorable short-term safety in young children with refractory classic juvenile PRP. Our findings support the potential role of TNF-α inhibitors as an effective alternative for severe pediatric PRP unresponsive to conventional therapies. Further prospective controlled trials are warranted to validate the long-term safety profile and optimize the dosage regimen for young pediatric populations.

## 1 Introduction

Pityriasis rubra pilaris (PRP) is a rare chronic inflammatory skin disorder, clinically characterized by follicular keratotic papules, orange-red scaly plaques, diffuse erythema, prominent palmoplantar keratoderma, and typical spared skin islands (1). Based on the Griffiths classification system, PRP is divided into six clinical subtypes. Classic juvenile PRP (type III) predominantly occurs in children, manifesting with an acute onset, rapid systemic generalization, and poor response to conventional treatment strategies (1, 2). Type III (classic juvenile) PRP usually affects children aged 5–10 years, presenting with follicular keratotic papules coalescing into extensive orange-red scaly plaques predominantly on the scalp, face and upper trunk, associated with orange palmoplantar keratoderma and characteristic islands of sparing. Most cases are sporadic and self-limited, with spontaneous remission within 1–2 years. This subtype needs differentiation from type IV PRP and CAPE. Type IV PRP manifests as well-demarcated localized plaques limited to bony prominences, lacking widespread facial and truncal lesions. CAPE, resulting from CARD14 variants, is often linked to a family history of psoriasis. Given the clinical overlap between CAPE and PRP, genetic assessment should be considered in patients with a positive psoriasis family history, which was not identified in our patient (3). Although oral retinoids are recognized as the first-line therapeutic agents for PRP, clinical responses vary significantly among individual patients (4). The clinical management of pediatric PRP remains a considerable challenge, especially for children under 5 years old. First-line conventional treatments mainly include topical corticosteroids, emollients and keratolytics. Moderate to severe pediatric PRP generally requires systemic interventions, such as oral retinoids, methotrexate, cyclosporine and phototherapy (5). However, most young patients fail to achieve complete lesion clearance with these regimens. Moreover, long-term application of conventional systemic agents is restricted by multiple safety concerns, including hepatotoxicity, myelosuppression, renal injury, growth retardation and teratogenicity (5, 6). In recent years, biologic agents targeting pro-inflammatory cytokines have been widely applied in the treatment of refractory adult PRP with satisfactory outcomes (7).

Adalimumab is a fully humanized anti-TNF-α monoclonal antibody. It has been officially approved for pediatric psoriasis and is commonly used off-label for a variety of refractory inflammatory dermatoses (9, 10). Accumulating studies have verified the efficacy of adalimumab in adult PRP, but relevant clinical data concerning its use in children under 5 years with PRP are still scarce (10). In this study, we describe a biopsy-confirmed case of classic juvenile PRP in a 4-year-old girl. The patient achieved rapid and sustained clinical remission with off-label adalimumab after multiple conventional therapies failed, thereby enriching the clinical evidence for biologic therapy in early-onset juvenile PRP.

## 2 Case report

A 4-year-7-month-old Chinese girl was admitted to the dermatology department due to a 20-day history of rapidly progressive generalized skin rash and severe pruritus. The patient had no remarkable past medical history, perinatal complications, or family history of atopic diseases, autoimmune disorders, hereditary dermatoses. No personal history of psoriasis was noted, and there was no family history of psoriasis in first- or second-degree relatives.

The skin lesions initially manifested as erythema and scaling around the mouth, periocular areas, palms and soles, and rapidly spread to the scalp, face, trunk and extremities within several days. The patient suffered from severe pruritus with a numerical rating scale (NRS) score of 7, resulting in frequent scratching behaviors and obvious sleep disturbances.

**Figure 1:**
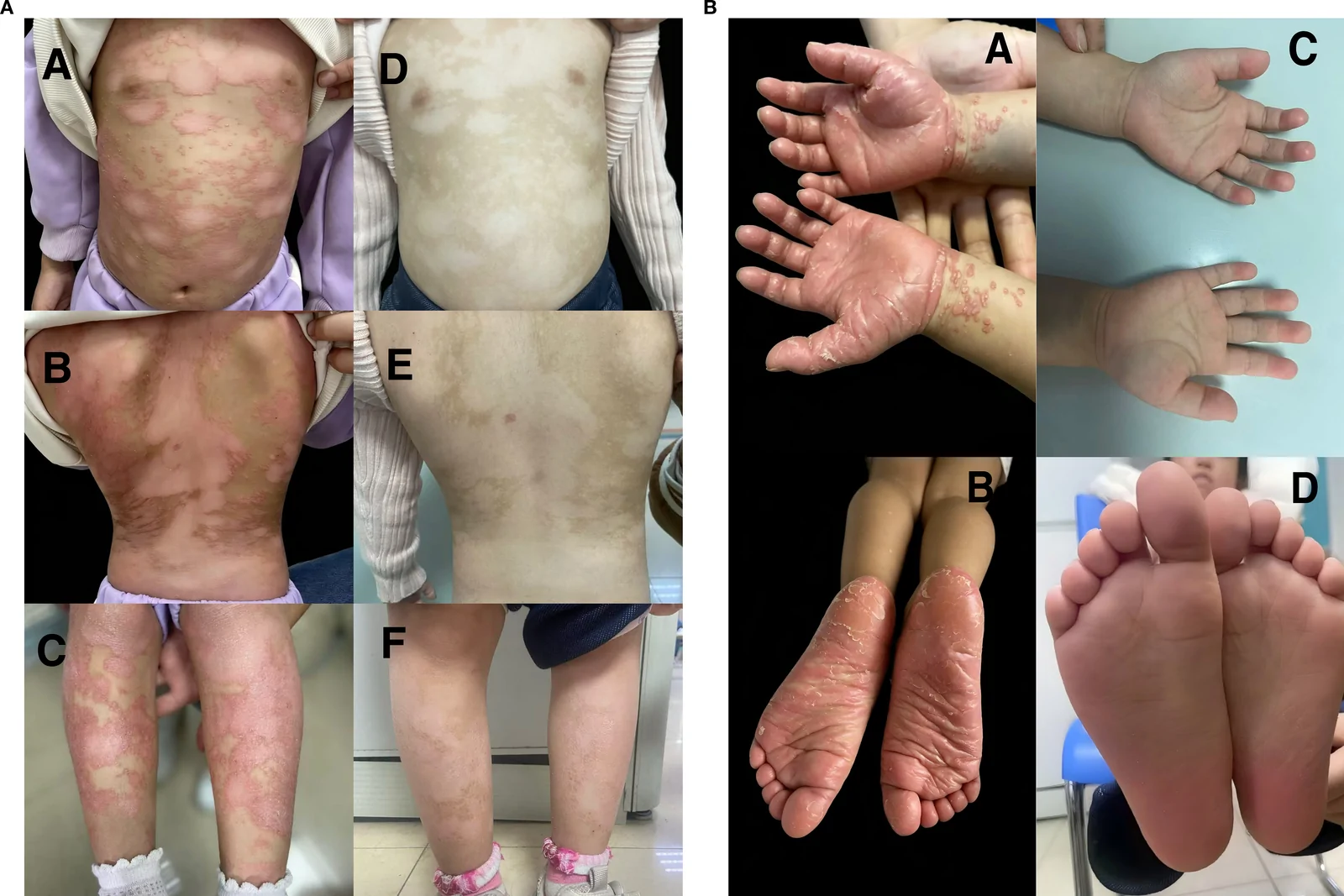
**(A)** Clinical presentation before and after adalimumab treatment. (A-C) Before treatment: Follicular keratotic papules fused into plaques with scales on the trunk and lower limbs. (D-F) After treatment: the rash subsided, leaving colored reduction spots. **(B)** before and after adalimumab treatment. (A, B) Before treatment: Marked palmoplantar keratosis with chapped palms. (C, D) After treatment: palmar and plantar skin completely returned to normal.

Dermatological physical examination revealed diffuse orange-red erythema covered with follicular keratotic papules and fine scales on the facial skin; thick and adherent scaling on the scalp surface; confluent erythematous scaly plaques distributed on the neck, axillae, trunk and limbs; and severe palmoplantar keratoderma accompanied by erythema and painful skin fissures (**Figure 1**). No systemic symptoms, such as fever, lymphadenopathy, or joint pain, were detected.

Routine blood parameters, liver and renal function, serum electrolytes and systemic inflammatory markers were all within normal reference ranges. Systemic infection screening yielded negative results, including tuberculosis interferon-gamma release assay, hepatitis B virus, hepatitis C virus and human immunodeficiency virus serological tests.

**Figure 2:**
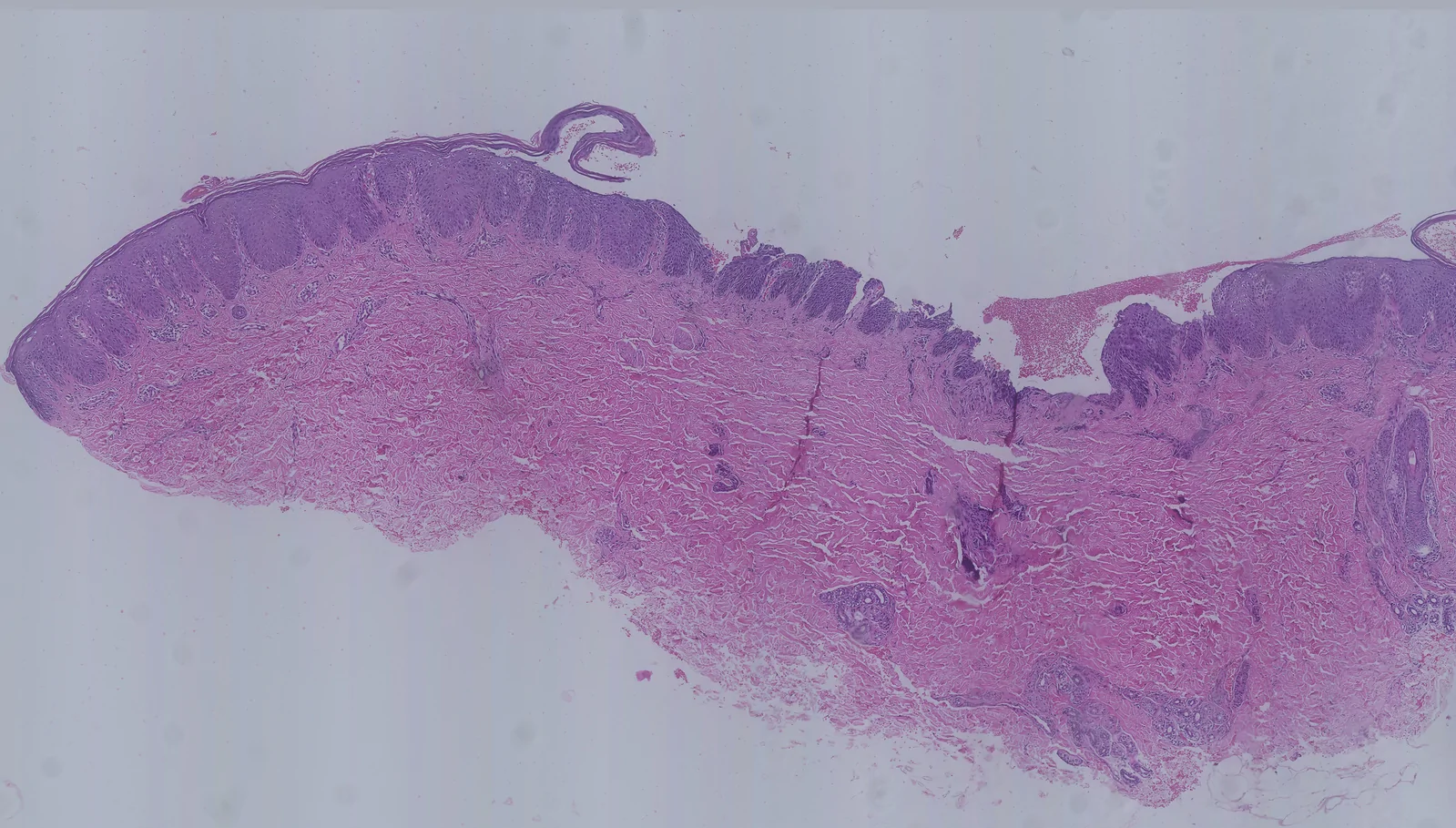
Skin histopathology (hematoxylin and eosin staining, original magnification ×10): Alternating orthokeratosis and parakeratosis, psoriasiform epidermal hyperplasia, and perivascular lymphocytic infiltration.

A 4-mm punch skin biopsy was performed on the erythematous plaque of the right thigh. Histopathological findings showed alternating orthokeratosis and parakeratosis, psoriasiform epidermal hyperplasia, dilated capillaries in the papillary dermis, and moderate perivascular lymphocytic infiltration in the superficial dermis (**Figure 2**). The above pathological features were consistent with the diagnostic criteria of PRP.

Before hospitalization, the patient had received sequential topical treatments including desonide cream, urea ointment and fluticasone propionate cream, combined with oral antihistamines and traditional Chinese medicine. Nevertheless, all these treatments failed to control disease progression.

After a comprehensive condition evaluation and written informed consent from the patient’s guardians, off-label adalimumab treatment was initiated. The patient’s body weight was 16 kg. The subcutaneous administration regimen was as follows: 20 mg at week 0, 20 mg at week 1, followed by a maintenance dose of 20 mg every two weeks, with a total of 8 subcutaneous injections.

The patient exhibited obvious clinical improvement shortly after the first adalimumab injection. At week 2, facial erythema was significantly alleviated, pruritus was markedly relieved with the NRS score decreased from 7 to 2, and no new skin lesions developed. At week 4, palmoplantar hyperkeratosis was obviously softened, and scaling on the trunk and extremities was greatly improved. Approximately 3 months after treatment initiation, all cutaneous lesions achieved complete clearance, with only mild residual hypopigmented macules. No adverse reactions were observed throughout the treatment period, including injection site irritation, secondary infection, or abnormal transaminase levels.

At the latest follow-up (8 months after the last adalimumab injection), the patient maintained complete remission without any signs of disease recurrence. Long-term regular outpatient follow-up is still ongoing.

## 3 Discussion

We reported a rare case of biopsy-proven classic juvenile PRP (Griffiths type III) in a 4-year-old girl. The patient achieved rapid and long-lasting clinical remission with off-label adalimumab after multiple conventional treatments proved ineffective. This case supplements the limited clinical data of anti-TNF-α biologic therapy in young children with PRP and provides a valuable reference for the individualized treatment of refractory early-onset PRP.

Classic juvenile PRP is characterized by acute onset, rapid systemic spread and prominent palmoplantar keratoderma (1, 2). In this case, the typical clinical manifestations, characteristic lesion distribution and specific histopathological changes facilitated the differential diagnosis from psoriasis, atopic dermatitis and other generalized erythrodermatoses.

The exact pathogenesis of PRP remains poorly defined. Aberrant immune activation and overexpression of the TNF-α signaling pathway play pivotal roles in the pathogenesis of PRP. Excessive TNF-α secretion induces keratinocyte hyperproliferation, dermal angiogenesis and massive inflammatory cell infiltration, thereby facilitating the formation and progression of PRP lesions (7, 8). Emerging evidence confirms involvement of the IL-23/Th17 immune axis in pityriasis rubra pilaris (PRP) pathogenesis. Nevertheless, the core molecular mechanism is incompletely characterized, resulting in prominent variability in clinical treatment outcomes. While the IL-23/Th17 pathway contributes to disease, TNF-α functions as a vital upstream mediator and constitutes an attractive therapeutic target (8). Existing therapeutic strategies for PRP include glucocorticoids, retinoids and conventional immunosuppressants, which frequently exhibit unsatisfactory and unstable clinical efficacy in many patients. Targeted biological agents have emerged as promising alternatives; recent data demonstrated satisfactory outcomes in pediatric PRP patients treated with secukinumab and other biologics (12). A broad spectrum of therapies, ranging from conventional systemic agents to biologics, can be utilized for PRP management, yet a subset of patients develops refractory disease unresponsive to standard regimens. Schmauch et al. further identified an IL-1-mediated molecular subgroup of PRP and confirmed the therapeutic benefit of IL-1 blockade in such refractory cases, advancing the understanding of PRP as an autoinflammatory keratinization disease (13). Refractory pediatric PRP, particularly in children under 5 years old, poses a substantial clinical challenge. Conventional systemic drugs, including oral retinoids, methotrexate and cyclosporine, are limited by insufficient efficacy and potential toxic side effects in young children (5, 14). Long-term use of these immunosuppressive and retinoid agents may cause irreversible damage, such as liver dysfunction, myelosuppression, renal damage and growth retardation (6, 15). In our case, multiple topical and adjuvant treatments failed to inhibit disease activity, which further reflected the intractable nature of severe early-onset PRP.

TNF-α inhibitors are among the earliest off-label biologic therapies for both classic and atypical juvenile PRP. As a fully humanized anti-TNF-α monoclonal antibody, adalimumab has been widely used in the treatment of pediatric psoriasis, juvenile idiopathic arthritis and other immune-mediated inflammatory diseases with confirmed efficacy and safety (9, 10, 16). To date, only one case of adalimumab for adolescent PRP has been documented in the literature (11). Our present report is the first to confirm the favorable effect of adalimumab in a 4-year-old pediatric patient, thereby expanding the applicable age range of TNF-α inhibitors for PRP treatment.

The adalimumab dosage adopted in this case (20 mg at weeks 0 and 1, followed by 20 mg every 2 weeks) refers to the standard dosage for children weighing 15–30 kg with pediatric psoriasis and juvenile idiopathic arthritis (10, 16). The patient achieved complete lesion clearance within 3 months and maintained relapse-free remission for 8 months after drug withdrawal, suggesting that a short-course intermittent adalimumab regimen is feasible for inducing long-term remission in children with classic juvenile PRP.

The short-term safety evaluation showed good tolerability of adalimumab in this young patient, without severe adverse events. Large-sample pediatric studies have confirmed that the overall safety profile of adalimumab is acceptable, and most adverse reactions are mild and controllable (16). Similarly, our patient had no obvious systemic complications during treatment and follow-up, which further verified the safety of short-term adalimumab application in young children with severe inflammatory skin diseases.

Several limitations of this case should be acknowledged. First, this is a single-center single case report with a limited 8-month follow-up period, which cannot draw definitive conclusions regarding long-term prognosis or delayed adverse reactions. Second, the optimal induction and maintenance dosage of adalimumab for juvenile PRP has not been verified by controlled clinical trials. Third, the extremely low incidence of classic juvenile PRP hinders the conduction of large-sample randomized controlled trials; therefore, standardized case reports are essential to accumulate clinical evidence for rare dermatoses.

## 4 Conclusion

In summary, we presented a case of biopsy-confirmed classic juvenile PRP in a 4-year-old girl. After failure of multiple conventional treatments, short-course off-label adalimumab therapy (8 injections in total) induced rapid complete remission of skin lesions, and the patient remained disease-free for 8 months after treatment discontinuation. Adalimumab exhibited rapid efficacy and good short-term tolerability in this young child with refractory PRP. This case provides new clinical evidence that TNF-α inhibitors are a safe and effective alternative for severe juvenile PRP in young children. Further multicenter longitudinal studies and long-term observational data are required to optimize the biologic treatment regimen and confirm its long-term safety in pediatric PRP.

## Data Availability

The original contributions presented in the study are included in the article/supplementary material. Further inquiries can be directed to the corresponding author.
